# Indacaterol on dyspnea in chronic obstructive pulmonary disease: a systematic review and meta-analysis of randomized placebo-controlled trials

**DOI:** 10.1186/1471-2466-13-26

**Published:** 2013-04-25

**Authors:** Jiangna Han, Lu Dai, Nanshan Zhong

**Affiliations:** 1 Department of Pneumology, Peking Union Medical College Hospital, Peking Union Medical College, Chinese Academy of Medical Sciences, Beijing, China; 2 Medical Affairs, Beijing Novartis Pharma Co., Ltd, BeijingChina; 3 State Key Laboratory of Respiratory Disease, First Affiliated Hospital, Guangzhou Medical College, Guangzhou, China; 4 Department of Pneumology, Peking Union Medical College Hospital, Shuaifuyuan No. 1, Beijing 100730, China

**Keywords:** Breathlessness, Baseline dyspnea index, Transition dyspnea index, Meta-analysis, COPD

## Abstract

**Background:**

Indacaterol is a novel, once-daily (od), inhaled, long-acting ß_2_-agonist bronchodilator for maintenance treatment of airflow limitation in patients with COPD. The aim of this study was to evaluate the efficacy of indacaterol on dyspnea, using available randomized placebo-controlled trials.

**Methods:**

A systematic search was made of MEDLINE, EMBASE, the Cochrane trials databases, and a manual search of journals. Randomized placebo-controlled trials of 12 weeks or more comparing indacaterol with placebo were reviewed, and eligible studies were included in a meta-analysis. The odds ratio (OR) for likelihood of achieving TDI score ≥ 1 after 12 weeks of treatment was used as an outcome measure to compare indacaterol to placebo.

**Results:**

Six trials were included in the analysis. Relative to placebo, the overall ORs for response were: indacaterol 75 μg od 1.784 (95% CI 1.282 to 2.482); indacaterol 150 μg od 2.149 (95% CI 1.746 to 2.645); and indacaterol 300 μg od 2.458 (95% CI 2.010 to 3.006). Overall OR for response in TDI tended to increase with higher indacaterol doses.

**Conclusions:**

Patients receiving indacaterol had clinically significant improvements in symptoms of dyspnea compared to placebo. Incremental benefits in TDI were observed with increasing doses. Indacaterol may provide patients and physicians with a useful treatment option in symptomatic patients with dyspnea.

## Background

The mortality, morbidity and economic burden of chronic obstructive pulmonary disease (COPD) is well documented [1,2]. Dyspnea as a cardinal symptom of COPD is a major cause of disability and anxiety associated with the disease, prompting the medical community to pursue effective treatments for the relief of dyspnea [3]. Recent guidelines recommend the regular use of inhaled long-acting bronchodilators to alleviate dyspnea in patients with symptomatic disease, with the addition of inhaled corticosteroids for patients who experience repeated exacerbations [1,2].

Indacaterol as a novel, once-daily (od), inhaled, long-acting ß_2_-agonist provides sustained bronchodilation for patients with moderate to severe COPD, with a rapid onset following the first dose [4,5]. It has been recently approved in Europe at doses of 150 μg and 300 μg od, in the United States at 75 μg od, and in China at 150 μg od for maintenance treatment of airflow limitation in patients with COPD. A number of randomized clinical trials suggest that indacaterol may improve dyspnea in patients with stable COPD, as indicated by changes in transition dyspnea index (TDI) [5-10]. However, all of these trials were statistically powered on forced expiratory volume in 1 second (FEV_1_) as a primary endpoint. The outcome measure of TDI was included as one of the secondary endpoints in these trials, and so might not have been adequately powered.

Therefore, we undertook a systematic review and meta-analysis of available randomized placebo-controlled studies to assess the efficacy of indacaterol on the important clinical outcome of dyspnea. The objectives of this meta-analysis were to combine data from existing randomized placebo-controlled trials, to use the number of patients achieving the minimum clinically important difference (MCID) for TDI score ≥ 1 as an outcome measure, and to evaluate the efficacy of once-daily indacaterol of licensed 75 μg, 150 μg, or 300 μg doses relative to placebo on dyspnea in patients with stable COPD.

## Methods

### Data sources and selection criteria

We identified published studies between January 2007 and May 2012 from MEDLINE, EMBASE, and the Cochrane Controlled Trials Register (CENTRAL) databases using the terms *indacaterol, long-acting ß*_*2*_*-agonist* AND *chronic obstructive pulmonary disease.* We performed a search of relevant files from the Novartis trials results database (http://www.novctrd.com/ctrdWebApp/clinicaltrialrepository/public/login.jsp). We also performed a manual search of references cited in published original and review articles, and in clinical practice guidelines. Trials published solely in abstract form were excluded because they contain preliminary and rudimentary information and may not provide enough details to allow full analysis. Two reviewers (JNH and LD) then independently screened potentially relevant trials from titles and abstracts. Using the full texts as necessary, the two reviewers independently identified eligible articles for full review. Finally, we reviewed eligible articles to determine whether they qualified for meta-analysis. Differences were resolved by discussion.

To be included, studies had to meet all the following criteria: a) target population of stable COPD consistent with American Thoracic Society/European Respiratory Society [11] or Global Initiative for Chronic Obstructive Lung Disease (GOLD) diagnostic criteria [12]; b) randomized placebo-controlled trials comparing indacaterol 75 μg, 150 μg, or 300 μg od with placebo; c) studies that followed patients for 12 weeks or more after randomization; d) studies that included assessment of dyspnea by transition dyspnea index (TDI) in outcome measures.

### Data extraction

Two reviewers independently read each article that met inclusion criteria and performed data extraction using a pre-designed data collection form. Missing data were obtained from the manufacturer. Disagreement and uncertainty were solved by discussion. Consensus was reached for all data. Data extracted from each article included: first author’s name and year of publication; study design; treatment arms; number of patients; treatment duration; baseline clinical characteristics including age, gender, smoking history, post-bronchodilator spirometry, and total score of the baseline dyspnea index (BDI). The outcome measure of TDI was recorded as total score and as the number of patients with TDI ≥ 1.

### Baseline dyspnea index (BDI) and transition dyspnea index (TDI)

Dyspnea is often measured using the BDI and TDI, a tool recommended by regulatory authorities for inclusion in clinical trials of treatments for COPD [13]. The BDI and TDI, as multidimensional instruments, each has three domains: functional impairment, magnitude of task, and magnitude of effort [14]. The BDI domains measure baseline dyspnea severity, and are rated from 0 (severe) to 4 (unimpaired) and summed to provide a BDI total score of 0 to 12, with a lower score indicating more severe dyspnea. The TDI domains measure change from the baseline dyspnea index (BDI) over time, rated on a scale of +3 (major improvement) to −3 (major deterioration). The TDI has been shown to be valid, reliable and responsive [14,15]. The minimum clinically important difference (MCID) for the TDI is an improvement from the BDI score of ≥1 unit [16,17].

### Statistical analyses

Odds ratio (OR) for likelihood of achieving TDI score ≥1 after 12 weeks of treatment was used as a measure to compare indacaterol relative to placebo. We calculated pooled ORs with the DerSimonian-Laird random effects model [18], usually regarded as more appropriate than other statistical approaches when potential heterogeneity is present between studies [19,20]. We performed separate analyses for indacaterol 75 μg versus placebo, indacaterol 150 μg versus placebo, and indacaterol 300 μg versus placebo. We calculated the 95% confidence intervals around the ORs, and assessed heterogeneity across studies with the chi-square test and *I*^*2*^ (p < 0.10, *I*^*2*^ > 25%). We then created forest plots of the individual studies and combined estimates. All analyses were performed with meta-analysis software (MetaAnalyst version beta 3.13, Tufts Medical Center, Boston, Massachusetts).

## Results

### Characteristics of included studies

Six studies met the inclusion criteria [5-9], the designs of which are summarized in Table 1. The studies varied from 12 to 52 weeks in duration, and so to permit comparison across trials, assessments at 12 weeks of treatment were used for the 52-week study [6] and for two 26-week studies [5,7]. We did not consider data from tiotropium, formoterol, and salmeterol arms for the current meta-analysis, since data for each of these arms would be provided from one study.

**Table 1:** Summary of study designs for the studies included in this meta-analysis

**Study**	**Design**	**Treatment arms**	**Number of patients**	**Treatment duration**
Donohue [5]	Phase III, randomized, double-blind (indacaterol and placebo) or open-label (tiotropium), placebo-controlled, parallel-group, multicentre	Indacaterol 150 μg od	1683 randomized;	26 weeks
		Indacaterol 300 μg od	1665 evaluable for efficacy	
		Tiotropium 18 μg od		
		Placebo		
Dahl [6]	Phase III, randomized, double-blind, double-dummy, placebo-controlled, parallel-group, multicentre	Indacaterol 300 μg od	1732 randomized;	52 weeks
		Indacaterol 600 μg od	1600 evaluable for efficacy	
		Formoterol 12 μg bid		
		Placebo		
Kornmann [7]	Phase III, randomized, double-blind, double-dummy, placebo-controlled, parallel-group, multicentre	Indacaterol 150 μg od	1002 randomized;	26 weeks
		Salmeterol 50 μg bid	998 evaluable for efficacy	
		Placebo		
Gotfried-1 [8]	Phase III, randomized, double-blind, placebo-controlled, parallel-group, in the United States	Indacaterol 75 μg od	323 randomized;	12 weeks
		Placebo	323 evaluable for efficacy	
Gotfried-2 [8]	Phase III, randomized, double-blind, placebo-controlled, parallel-group, in the United States	Indacaterol 75 μg od	318 randomized;	12 weeks
		Placebo	317 evaluable for efficacy	
Kinoshita [9]	Phase III, randomized, double-blind, placebo-controlled, parallel-group, in six Asian areas	Indacaterol 150 μg od	347 randomized;	12 weeks
		Indacaterol 300 μg od	347 evaluable for efficacy	
		Placebo		

od = once-daily; bid = twice-daily.

Table 2 illustrates patient populations and baseline characteristics. Of the six studies providing the data for the meta-analysis, four had similar inclusion criteria [5-7,9], recruiting male and female patients aged ≥40 years with a clinical diagnosis of moderate-to-severe COPD as per the GOLD 2005 criteria and a smoking history of ≥20 pack-years. Postbronchodilator FEV_1_ was to be <80% and ≥30% predicted and post-bronchodilator FEV_1_/forced vital capacity <70% [5-7,9]. The two identical indacaterol 75 μg trials enrolled patients with moderate-to-severe COPD defined at that time using GOLD 2008 criteria, aged ≥40 years and with a smoking history of ≥10 pack-years [8].

**Table 2:** Patient characteristics at baseline in the individual studies

**Study**	**Treatment arms**	**Patients, n**^**a**^	**Age, years**	**Male, n (%)**	**Smoking history, pack-years**	**FEV**_**1**_**, L**^**b**^	**FEV**_**1**_**, % pred**^**b**^	**FEV**_**1**_**/FVC, %**^**b**^	**BDI total score**^**c**^
Donohue [5]	Indacaterol 150 μg od	416	63.4 (40, 87)	259 (62.3)	48.3 (20, 150)	1.52 (0.62, 3.45)	56.1( 29.3, 116.6)	53.0 (24.4, 69.7)	6.56 (0, 12)
	Indacaterol 300 μg od	416	63.3 (40, 88)	263 (63.2)	50.8 (13, 208)	1.53 (0.57, 3.14)	56.3 (21.3, 90.0)	52.6 (25.7, 69.5)	6.52 (0, 12)
	Tiotropium 18 μg od	415	64.0 (41, 85)	269 (64.8)	50.0 (20, 180)	1.45 (0.48, 3.00)	53.9 (23.6, 132.3)	52.7 (24.7, 72.6)	6.57 (0, 12)
	Placebo	418	63.6 (41, 84)	255 (61.0)	49.7 (20, 156)	1.51 (0.53, 2.98)	56.1 (28.4, 95.1)	53.4 (24.0, 69.9)	6.39 (0, 12)
Dahl [6]	Indacaterol 300 μg od	437	63.9 (40, 87)	351 (80.3)	48.6 (20, 600)	1.48 (0.44, 2.95)	52.8 (23.5, 101.4)	51.1 (27.7, 90.1)	6.62 (0, 12)
	Indacaterol 600 μg od	425	62.9 (40, 87)	327 (76.9)	53.6 (20, 900)	1.48 (0.55, 2.91)	51.6 (24.0, 84.2)	51.1 (15.8, 84.4)	6.57 (0, 12)
	Formoterol 12 μg bid	434	63.6 (40, 84)	348 (80.2)	49.0 (20, 800)	1.50 (0.59, 3.25)	52.9 (20.8, 100.5)	51.3 (23.0, 96.5)	6.46 (1, 12)
	Placebo	432	63.2 (41, 90)	352 (81.5)	53.3 (20, 900)	1.52 (0.58, 3.09)	52.9 (17.6, 96.3)	52.1 (21.5, 80.0)	6.52 (0, 12)
Kornmann [7]	Indacaterol 150 μg od	330	63.2 (41, 85)	238 (72.1)	39.6 (20, 120)	1.48 (0.63, 2.93)	53.9 (30.0, 104.4)	53.5 (23.5, 76.8)	6.74 (0, 12)
	Salmeterol 50 μg bid	333	63.4 (41, 86)	249 (74.8)	40.0 (20, 147)	1.48 (0.43, 3.18)	53.1 (17.9, 93.4)	52.2 (18.0, 82.2)	6.68 (0, 12)
	Placebo	335	63.9 (42, 89)	258 (77.0)	41.0 (20, 159)	1.46 (0.47, 3.20)	53.0 (12.3, 91.0)	52.7 (20.2, 90.9)	6.63 (0, 12)
Gotfried-1 [8]	Indacaterol 75 μg od	163	64.0 (44, 85)	89 (54.6)	52.9 (10, 150)	1.49 (0.57, 2.92)	53.7 (30.3, 77.3)	53.1 (31.8, 68.3)	6.40 (2, 12)
	Placebo	160	64.1 (40, 90)	87 (54.4)	51.2 (10, 148)	1.46 (0.62, 2.77)	53.3 (29.5, 78.7)	51.6 (25.6, 69.1)	5.81 (1, 12)
Gotfried-2 [8]	Indacaterol 75μg od	159	61.3 (40, 82)	83 (52.2)	52.4 (11, 180)	1.59 (0.65, 3.39)	55.7 (29.7, 79.3)	52.4 (22.9, 68.6)	6.01 (0, 12)
	Placebo	158	61.5 (42, 86)	89 (56.0)	52.4 (10, 204)	1.52 (0.56, 3.00)	53.5 (30.3, 79.4)	52.6 (28.8, 69.1)	6.15 (1, 11)
Kinoshita [9]	Indacaterol 150 μg od	114	66.4 (46, 83)	110 (96.5)	51.7 (20, 196)	1.46 (0.70, 2.50)	55.2 (30.0, 79.0)	50.3 (27.0, 69.0)	7.53 (1, 12)
	Indacaterol 300 μg od	116	67.1 (48, 86)	113 (97.4)	54.0 (20, 150)	1.41 (0.68, 2.92)	53.7 (30.0, 79.0)	48.7 (27.0, 69.0)	7.67 (1, 12)
	Placebo	117	66.5 (40, 88)	112 (95.7)	49.7 (20, 160)	1.38 (0.55, 2.37)	52.3 (30.0, 77.0)	47.7 (28.0, 69.0)	7.35 (1, 12)

Data are mean (min, max) for age, smoking history, FEV_1_, FEV_1_ % predicted, and FEV_1_/FVC% unless stated otherwise.

a Number of patients analysed for efficacy (ITT, modified ITT or FAS).

b Post-bronchodilator spirometry.

c Values for patients who provided data at Week 12.

### Dyspnea – BDI and TDI

In all trials included in the meta-analysis, dyspnea was measured at baseline using the BDI. As illustrated in Table 2, patients had moderate severity of dyspnea at baseline with mean BDI total scores ranging from 5.81 to 7.67. After 12 weeks of treatment, dyspnea was measured using the TDI (Table 3), which captured changes from baseline. Data are presented as mean TDI total scores and as the number of patients with TDI score ≥1 unit. Results were analyzed for the number of patients responding with a change of TDI equal to or greater than the MCID (‘responder analysis’) (Table 3).

**Table 3:** Indacaterol on dyspnea as measured by the TDI

**Study**	**Treatment arms**	**TDI at week 12**		
		**Patients, n**^**a**^	**TDI total score**^**b**^	**Number of patients with TDI** ≥**1**
Donohue [5]	Indacaterol 150 μg od	355	2.09 (-8, 9)	209
	Indacaterol 300 μg od	363	2.40 (-9, 9)	239
	Tiotropium 18 μg od	360	1.89 (-6, 9)	198
	Placebo	326	1.19 (-6, 9)	138
Dahl [6]	Indacaterol 300 μg od	364	2.11 (-9, 9)	229
	Indacaterol 600 μg od	348	2.10 (-7, 9)	202
	Formoterol 12 μg bid	359	1.64 (-9, 9)	190
	Placebo	343	0.87 (-9, 9)	138
Kornmann [7]	Indacaterol 150 μg od	303	2.37 (-6, 9)	182
	Salmeterol 50 μg bid	296	1.60 (-6, 9)	152
	Placebo	286	0.87 (-9, 9)	113
Gotfried-1 [8]	Indacaterol 75 μg od	150	1.36 (-6, 9)	73
	Placebo	150	0.12 (-9, 9)	48
Gotfried-2 [8]	Indacaterol 75 μg od	148	1.25 (-7, 9)	69
	Placebo	149	0.83 (-6, 9)	53
Kinoshita [9]	Indacaterol 150 μg od	108	2.16 (-3, 9)	66
	Indacaterol 300 μg od	107	2.04 (-3, 9)	58
	Placebo	102	0.88 (-6, 9)	40

a Number of patients evaluated for TDI total score at week 12 and included in the responder analysis.

b Values are mean (min, max) of raw and unadjusted TDI total score.

### Indacaterol 75 μg versus placebo

**Figure 1:**
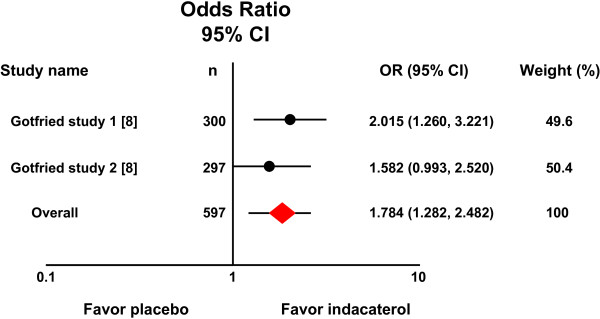
**Forest plot of odds ratios and 95% confidence intervals.** Relative to placebo, patients receiving indacaterol 75 μg once daily are more likely to achieve TDI score equal to or greater than one unit.

Two randomized placebo-controlled studies had identical entry criteria and study designs, which compared indacaterol 75 μg once daily with placebo after 12 weeks of treatment [8]. A meta-analysis that combined the two studies produced a pooled OR estimate of 1.784 (95% CI 1.282–2.482) with no evidence of heterogeneity (*P =* 0.474, I^2^ = 0.000), indicating that relative to placebo, patients receiving indacaterol 75 μg are more likely to achieve TDI score ≥1 after 12 weeks of treatment. Figure 1 shows a forest plot of OR estimates from these studies.

### Indacaterol 150 μg versus placebo

**Figure 2:**
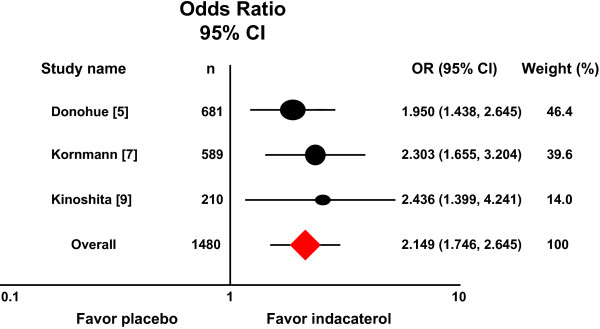
**Meta-analysis of three randomized trials compared indacaterol 150 μg once daily with placebo.** Relative to placebo, patients receiving indacaterol 150 μg once daily are more likely to achieve TDI score equal to or greater than one unit.

Three randomized placebo-controlled trials compared indacaterol 150 μg once daily with placebo [5,7,9]. A meta-analysis that combined the three studies produced a pooled OR estimate of 2.149 (95% CI 1.746–2.645) with no evidence of heterogeneity (*P =* 0.686, I^2^ = 0.000), favoring patients who received indacaterol 150μg once daily. Figure 2 shows a forest plot of OR estimates from these studies.

### Indacaterol 300 μg versus placebo

**Figure 3:**
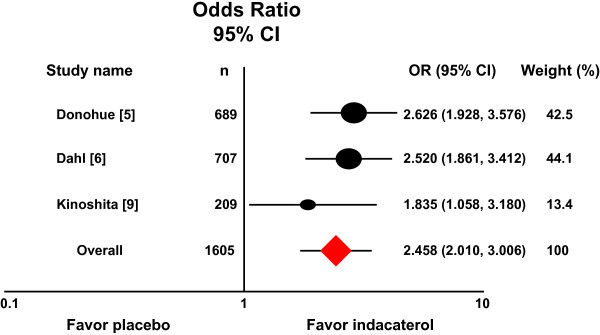
**Meta-analysis of three randomized trials compared indacaterol 300 μg once daily with placebo.** Relative to placebo, patients receiving indacaterol 300 μg once daily are more likely to achieve TDI score equal to or greater than one unit.

Three trials compared indacaterol 300 μg once daily with placebo [5,6,9]. Figure 3 shows a forest plot of OR estimates from these studies. The combined OR estimate was 2.458 (95% CI 2.010–3.006) with no evidence of heterogeneity (*P =* 0.525, I^2^ = 0.000), again favoring patients who received indacaterol 300 μg once daily.

## Discussion

The present meta-analyses combined data from existing randomized placebo-controlled trials, using number of patients achieving the minimum clinically important difference (MCID) for TDI score ≥1 as an outcome measure, and evaluated the efficacy of once-daily indacaterol on TDI scores in patients with stable COPD. A favorable effect was consistently obtained for indacaterol over placebo: 75 μg od OR 1.784 (95% CI 1.282 to 2.482); 150 μg od OR 2.149 (95% CI 1.746 to 2.645); and 300 μg od OR 2.458 (95% CI 2.010 to 3.006). A trend of increasing patient benefit was observed as indacaterol doses increased.

Recent guidelines recommend regular use of bronchodilators, such as indacaterol, as a long-term maintenance treatment of airflow limitation in COPD [1]. The extent to which indacaterol improves airflow in patients with COPD is well studied [4,5,21-24]. In these phase III studies, indacaterol provided 24-h bronchodilation on once-daily dosing with an effect that was sustained during treatment for up to one year. However, from the patients’ viewpoint, it may be more important to know whether this therapy improves dyspnea associated with daily activities, and these meta-analyses of available randomized placebo-controlled trials comparing indacaterol with placebo allows us to examine the efficacy of indacaterol on this important patient outcome. In all trials included in the meta-analyses, dyspnea was assessed using validated multidimensional instruments of BDI and TDI that are widely used to measure treatment effects in COPD [13-17]. When data were analyzed for a pre-determined outcome, i.e. the number of patients responding with a change of TDI equal to or greater than the minimum clinically important difference MCID (a ‘responder analysis’), a favorable effect was obtained for all three doses of indacaterol. The overall OR estimates of the meta-analyses are largely consistent with the results from the individual studies, in which the ORs for patients achieving a MCID in the indacaterol group compared with the placebo group varied from 1.582 to 2.015 for the 75 μg dose (Figure 1) [8], 1.950 to 2.303 for the 150 μg dose (Figure 2) [5,7], and 2.520 to 2.626 for the 300 μg dose (Figure 3) [5,6], with a exception of the Asian study [9]. It becomes apparent that patients receiving indacaterol had clinically significant improvements in symptoms of dyspnea compared to placebo over 12 weeks of treatment.

The overall odds ratio for response in TDI appeared to vary depending on indacaterol doses, and tended to increase with increasing indacaterol doses. Additionally, the percentage of patients who exceeded the one unit of the MCID in TDI varied in different indacaterol doses. The percentage in the analysis of indacaterol 75 μg once daily was 48%, compared with 34% in placebo. Compared to placebo, a higher percentage of patients achieved the MCID with the indacaterol 150 μg (60% versus 41%) and indacaterol 300 μg doses (63% versus 41%). Renard and his colleagues recently performed a model based analysis of the bronchodilatory dose response to indacaterol in patients with COPD [25]. The analysis demonstrated that indacaterol dosages of 75 μg once daily and above achieved minimal clinically important improvements in predicted trough FEV_1_ response, although dosages of 150 μg and 300 μg once daily provided optimum bronchodilation. The analysis also demonstrated that disease severity, as determined by FEV_1_, significantly affected dose response, suggesting that higher doses may be required in patients with more severe COPD to achieve optimal reduction of dyspnea.

In six trials included in the meta-analysis (n=5405), patients with COPD who received indacaterol 75 μg, or 150 μg, or 300 μg od had a significantly higher trough FEV_1_ than placebo after at least 12 weeks, with indacaterol increasing trough FEV_1_ by 120ml to 200 ml over placebo at week 12 [5-9]. The overall improvement in FEV_1_, though modest, may be sufficient to decrease the extent of hyperinflation which contributes to the sensation of dyspnea [26-29]. A better indicator of the effect of a bronchodilator on hyperinflation (and therefore the sensation of dyspnea) is perhaps inspiratory capacity. One could hypothesize that the prolonged bronchodilation observed with indacaterol would be associated with reductions of air trapping, and therefore reductions in hyperinflation, which would then be reflected in improvements in the sensation of dyspnea. There are relatively few indacaterol studies that included both an assessment of inspiratory capacity and of dyspnea, however, two short-term exercise studies comparing the 300 μg dose of indacaterol with placebo have been published [30,31]. The BDI/TDI was included in one of the studies, an improvement of 182 ml in resting inspiratory capacity after 14 days (p<0.05 vs. placebo) was associated with a change of TDI total score of +3.33 (p<0.01 vs. placebo) [31], providing indirect evidence to support the hypothesis.

## Conclusions

Once daily indacaterol provides clinically significant improvements in dyspnea compared with placebo after 12 weeks of treatment. Incremental benefits in TDI were observed with increasing doses of indacaterol. Indacaterol may provide patients and physicians with a useful treatment option in symptomatic patients with dyspnea. Further studies, that investigate the relationship between disease severity as determined by FEV_1_ and dose response to indacaterol in terms of dyspnea, are needed to specifically address the question whether higher doses are required in patients with more severe COPD to achieve optimal reduction of dyspnea.

## Abbreviations

BDI: Baseline dyspnea index; COPD: Chronic obstructive pulmonary disease; FEV1: Forced expiratory volume in 1 second; FVC: Forced vital capacity; GOLD: Global initiative for obstructive lung disease; MCID: Minimal clinically important difference; TDI: Transition dyspnea index; RCTs: Randomized controlled trials.

## Competing interests

JNH has no conflict of interests to declare. LD is an employee of Novartis China. NSZ has no conflict of interests to declare.

## Authors’ contributions

JNH, LD and NSZ were involved in the concept and design of this article and the interpretation of the data. JNH was responsible for analysis of data. All authors were participated in all stages of developing the manuscript. All authors revised the manuscript critically for important intellectual content, and gave their final approval of the version to be published.

## Pre-publication history

The pre-publication history for this paper can be accessed here:

http://www.biomedcentral.com/1471-2466/13/26/prepub

## References

[B1] Global Initiative for Obstructive Lung Disease (GOLD) Global strategy for the diagnosis, management, and prevention of chronic obstructive pulmonary disease 2011 http://www.goldcopd.org/Guidelines/guidelines-gold-summary-2011.html

[B2] Qaseem A Wilt TJ Weinberger SE Hanania NA Criner G van der Molen T Marciniuk DD Denberg T Schünemann H Wedzicha W MacDonald R Shekelle P Diagnosis and management of stable chronic obstructive pulmonary disease: a clinical practice guideline update from the American College of Physicians, American College of Chest Physicians, American Thoracic Society, and European Respiratory Society Ann Intern Med 2011 155 179 191 21810710 10.7326/0003-4819-155-3-201108020-00008

[B3] Mahler DA Selecky PA Harrod CG Benditt JO Carrieri-Kohlman V Curtis JR Manning HL Mularski RA Varkey B Campbell M Carter ER Chiong JR Ely EW Hansen-Flaschen J O’Donnell DE Waller A American College of Chest Physicians consensus statement on the management of dyspnea in patients with advanced lung or heart disease Chest 2010 137 674 691 10.1378/chest.09-1543 20202949

[B4] Balint B Watz H Amos C Owen R Higgins M Kramer B Onset of action of indacaterol in patients with COPD: Comparison with salbutamol and salmeterol-fluticasone Int J Chron Obstruct Pulmon Dis 2010 5 311 318 20856830 10.2147/copd.s12120 PMC2939686

[B5] Donohue JF Fogarty C Lötvall J Mahler DA Worth H Yorgancioglu A Iqbal A Swales J Owen R Higgins M Kramer B Once-daily bronchodilators for chronic obstructive pulmonary disease Am J Respir Crit Care Med 2010 182 155 162 10.1164/rccm.200910-1500OC 20463178

[B6] Dahl R Chung KF Buhl R Magnussen H Nonikov V Jack D Bleasdale P Owen R Higgins M Kramer B Efficacy of a new once-daily long-acting inhaled beta2-agonist indacaterol versus twice-daily formoterol in COPD Thorax 2010 65 473 479 10.1136/thx.2009.125435 20522841

[B7] Kornmann O Dahl R Centanni S Dogra A Owen R Lassen C Kramer B Once-daily indacaterol versus twice-daily salmeterol for COPD: a placebo-controlled comparison Eur Respir J 2011 37 273 279 10.1183/09031936.00045810 20693243

[B8] Gotfried MH Kerwin EM Lawrence D Lassen C Kramer B Efficacy of indacaterol 75 μg once-daily on dyspnea and health status: results of two double-blind, placebo-controlled 12-week studies COPD 2012 9 1 8 10.3109/15412555.2012.651935 23020650

[B9] Kinoshita M Lee SH Hang LW Ichinose M Hosoe M Okino N Prasad N Kramer B Fukuchi Y Efficacy and safety of indacaterol 150 and 300 μg in chronic obstructive pulmonary disease patients from six Asian areas including Japan: a 12-week, placebo-controlled study Respirology 2012 17 379 389 10.1111/j.1440-1843.2011.02107.x 22122202

[B10] Kerwin EM Gotfried MH Lawrence D Lassen C Kramer B Efficacy and tolerability of indacaterol 75 μg once daily in patients aged ≥40 years with chronic obstructive pulmonary disease: results from 2 double-blind, placebo-controlled 12-week studies Clin Ther 2011 33 1974 1984 10.1016/j.clinthera.2011.11.009 22177371

[B11] Celli BR MacNee W Agusti A Anzueto A Berg B Buist AS Calverley PMA Chavannes N Dillard T Fahy B Fein A Heffner J Lareau S Meek P Martinez F McNicholas W Muris J Austegard E Pauwels R Rennard S Rossi A Siafakas N Tiep B Vestbo J Wouters E ZuWallack R Standards for the diagnosis and treatment of patients with COPD: a summary of the ATS/ERS position paper Eur Respir J 2004 23 932 946 10.1183/09031936.04.00014304 15219010

[B12] Global Initiative for Obstructive Lung Disease (GOLD) Global strategy for the diagnosis, management, and prevention of chronic obstructive pulmonary disease 2005 http://www.goldcopd.org/search.html?q=GOLD+2005+update&user

[B13] Jones P Lareau S Mahler DA Measuring the effects of COPD on the patient Respir Med 2005 99 Suppl B S11 S18 16236492 10.1016/j.rmed.2005.09.011

[B14] Mahler DA Weinberg DH Wells CK Feinstein AR The measurement of dyspnea. Contents, interobserver agreement, and physiologic correlates of two new clinical indexes Chest 1984 85 751 758 10.1378/chest.85.6.751 6723384

[B15] Witek TJ Jr Mahler DA Meaningful effect size and patterns of response of the transition dyspnea index J Clin Epidemiol 2003 56 248 255 10.1016/S0895-4356(02)00589-9 12725879

[B16] Witek TJ Jr Mahler DA Minimal important difference of the transition dyspnea index in a multinational clinical trial Eur Respir J 2003 21 267 272 10.1183/09031936.03.00068503a 12608440

[B17] Mahler DA Witek TJ Jr The MCID of the transition dyspnea index is a total score of one unit COPD 2005 2 99 103 10.1081/COPD-200050666 17136969

[B18] DerSimonian R Laird N Meta-analysis in clinical trials Contr Clin Trials 1986 7 177 188 10.1016/0197-2456(86)90046-2 3802833

[B19] Mosteller F Colditz GA Understanding research synthesis (meta-analysis) Annu Rev Publ Health 1996 17 1 23 10.1146/annurev.pu.17.050196.000245 8724213

[B20] Bates MN Khalakdina A Pai M Chang L Lessa F Smith KR Risk of tuberculosis from exposure to tobacco smoke: a systematic review and meta-analysis Arch Intern Med 2007 167 335 342 10.1001/archinte.167.4.335 17325294

[B21] Barnes PJ Pocock SJ Magnussen H Iqbal A Kramer B Higgins M Lawrence D Integrating indacaterol dose selection in a clinical study in COPD using an adaptive seamless design Pulm Pharmacol Ther 2010 23 165 171 10.1016/j.pupt.2010.01.003 20080201

[B22] Vogelmeier C Ramos-Barbon D Jack D Piggott S Owen R Higgins M Kramer B Indacaterol provides 24-hour bronchodilation in COPD: a placebo-controlled blinded comparison with tiotropium Respir Res 2010 11 135 10.1186/1465-9921-11-135 20920365 PMC2964613

[B23] Magnussen H Verkindre C Jack D Jadayel D Henley M Woessner R Higgins M Kramer B Indacaterol once-daily is equally effective dosed in the evening or morning in COPD Respir Med 2010 104 1869 1876 10.1016/j.rmed.2010.08.010 20850959

[B24] Chapman KR Rennard SI Dogra A Owen R Lassen C Kramer B Long-term safety and efficacy of indacaterol, a long-acting ß_2_-agonist, in subjects with COPD: a randomized, placebo-controlled study Chest 2011 140 68 75 10.1378/chest.10-1830 21349928

[B25] Renard D Looby M Kramer B Lawrence D Morris D Stanski DR Characterization of the bronchodilatory dose response to indacaterol in patients with chronic obstructive pulmonary disease using model-based approaches Respir Res 2011 12 54 10.1186/1465-9921-12-54 21518459 PMC3102616

[B26] Cooper CB Airflow obstruction and exercise Respir Med 2009 103 325 334 10.1016/j.rmed.2008.10.026 19071004

[B27] Hanania NA Donohue JF Pharmacologic interventions in chronic obstructive pulmonary disease Proc Am Thorac Soc 2007 4 526 534 10.1513/pats.200701-016FM 17878465

[B28] Malerba M Radaeli A Morjaria JB Therapeutic potential for novel untra long-acting ß_2_-agonists in the management of COPD: biological and pharmacological aspects Drug Discov Today 2012 17 496 504 10.1016/j.drudis.2011.11.002 22119310

[B29] Fuso L Mores N Valente S Malerba M Montuschi P Long-acting beta-agonists and their association with inhaled corticosteroids in COPD Curr Med Chem 2013 Epub ahead of print 10.2174/0929867311320120003 23409722

[B30] O’Donnell DE Casaburi R Vincken W Puente-Maestu L Swales J Lawrence D Kramer B Effect of indacaterol on exercise endurance and lung hyperinflation in COPD Respir Med 2011 105 1030 1036 10.1016/j.rmed.2011.03.014 21498063

[B31] Beeh KM Wagner F Khindri S Drollmann AF Effect of indacaterol on dynamic lung hyperinflation and breathlessness in hyperinflated patients with COPD COPD 2011 8 340 345 10.3109/15412555.2011.594464 21793716

